# Mitigation of age-dependent accumulation of defective mitochondrial genomes

**DOI:** 10.1073/pnas.2119009119

**Published:** 2022-07-26

**Authors:** Pei-I Tsai, Ekaterina Korotkevich, Patrick H. O’Farrell

**Affiliations:** ^a^Department of Biochemistry and Biophysics, University of California, San Francisco, CA, 94158

**Keywords:** mitochondria, mtDNA, heteroplasmy, aging, mutations

## Abstract

Unknown processes promote the accumulation of mitochondrial DNA (mtDNA) mutations during aging. Accumulation of defective mitochondrial genomes is thought to promote the progression of heteroplasmic mitochondrial diseases and degenerative changes with natural aging. We used a heteroplasmic *Drosophila* model to test 1) whether purifying selection acts to limit the abundance of deleterious mutations during development and aging, 2) whether quality control pathways contribute to purifying selection, 3) whether activation of quality control can mitigate accumulation of deleterious mutations, and 4) whether improved quality control improves health span. We show that purifying selection operates during development and growth but is ineffective during aging. Genetic manipulations suggest that a quality control process known to enforce purifying selection during oogenesis also suppresses accumulation of a deleterious mutation during growth and development. Flies with nuclear genotypes that enhance purifying selection sustained higher genome quality, retained more vigorous climbing activity, and lost fewer dopaminergic neurons. A pharmacological agent thought to enhance quality control produced similar benefits. Importantly, similar pharmacological treatment of aged mice reversed age-associated accumulation of a deleterious mtDNA mutation. Our findings reveal dynamic maintenance of mitochondrial genome fitness and reduction in the effectiveness of purifying selection during life. Importantly, we describe interventions that mitigate and even reverse age-associated genome degeneration in flies and in mice. Furthermore, mitigation of genome degeneration improved well-being in a *Drosophila* model of heteroplasmic mitochondrial disease.

## Significance

In contrast to the orderly segregation of nuclear DNA, mitochondrial genomes compete for replication and segregation. The abundance of mutants that emerges in a cell depends on their success in this competition. We show that quality control mechanisms put deleterious mutations at a disadvantage but that these mechanisms become ineffective during aging. The resulting rise in mutant genomes, which compromises vigor, can be suppressed using genetic backgrounds that enhance quality control. Feeding kinetin to adult *Drosophila* or mice also reduced the load of mutant mitochondrial genomes. This pharmacological reversal of the age-associated deterioration of mitochondrial DNA quality suggests possible therapies to alleviate mitochondrial diseases and normal aging.

Unlike nuclear genotype, which is largely stable during one’s lifetime, when genetically distinct mitochondrial genomes co-reside (heteroplasmy), their relative proportions shift during growth, development, and aging. This shift is not random (1–7). Mutant mitochondrial DNA (mtDNA) variants accumulate during aging and in the progression of some mitochondrial diseases (1–5). Stereotyped changes in abundance of particular alleles in different tissues in human and mouse indicate that selective forces favor different mitochondrial genomes (3, 6, 7). However, despite the importance of mitochondrial function to health and well-being, we have limited understanding of the processes underlying the accumulation of mitochondrial mutations with age.

The nuclear genome encodes mechanisms of quality control that survey the function of mitochondria and eliminate or compromise the proliferation of defective mitochondria (8, 9). Two described mechanisms use PINK1, the product of a gene discovered as one of the causes of early-onset familial Parkinson’s disease, as a sensor of mitochondrial function. PINK1 accumulates on the surface of mitochondria having a reduced membrane potential, and its kinase activity in this location signals several downstream events (10–15). In one pathway defined largely in a cell culture model, PINK1 activates PARKIN, the product of another Parkinson’s disease gene, which then triggers elimination of compromised mitochondria by mitophagy (16). In a second pathway, acting in the *Drosophila* female germ line (17, 18), PINK1 acts in a PARKIN-independent pathway that targets a protein called Larp to inhibit its role in promoting biogenesis of the mitochondria (18) (*SI Appendix*, Fig. S2).

Since deleterious mtDNA mutations compromise electron transport of mitochondria, it seems that quality control would put genomes carrying such mutations at a disadvantage, creating a purifying selection that leads to their elimination. However, this outcome is far from certain. Various factors such as the sharing of gene products among mitochondria as a result of dynamic fission and fusion could mask the consequences of heteroplasmic mutations, shielding them from quality control. Indeed, a number of studies suggest a contrast between the germ line, which exhibits purifying selection, and adult somatic tissues, which often show accumulation of mutations. Studies in *Drosophila* show that purifying selection acting in the female germ line eliminates deleterious mutations in a few generations (18–21). Genetic dissection revealed that this purifying selection depends on the PINK1/LARP pathway of quality control (18). On the other hand, an elegant study that induced heteroplasmic deletions in the flight muscle of the adult fly detected no substantial indications of purifying selection unless additional stressors were introduced (22). Furthermore, a detailed study in *Drosophila* carrying a proofreading defective mitochondrial DNA polymerase (mutator line) showed that adult flies accumulate a spectrum of mutations biased toward deleterious mutations (23). Since deleterious mutations would potentially be removed by purifying selection, this finding argues either that it was not operating or that it was opposed by a stronger selection favoring deleterious mutations (23). Similarly, studies in mouse suggest a discordance between germ line and soma. When mutations in the mitochondrial genome that were introduced in a mutator line were passed through subsequent generations in a wild-type background, there was selective elimination of deleterious mutations, a strong signal of purifying selection (7). In contrast, zygotically accumulated mutations in the mutator mouse exhibited high levels of deleterious mutations, suggesting a lack of purifying selection in the soma. Furthermore, Parkin mutant mice did not show a significant increase in mutations in adult wild-type or mutator mice, suggesting that Parkin-dependent quality control does not contribute to purifying selection (16). While these studies suggest major changes in the efficiency of purifying selection, other studies have suggested continued purifying selection in some circumstances. For example, adult human T cells of mitochondrial disease patients show exceptionally low heteroplasmy levels, suggesting cell type-specific action of purifying selection (24). We sought to measure purifying selection and to understand the nature of quality control in the soma during growth, development, and aging using an experimental model developed in *Drosophila*.

**Fig. 1.**
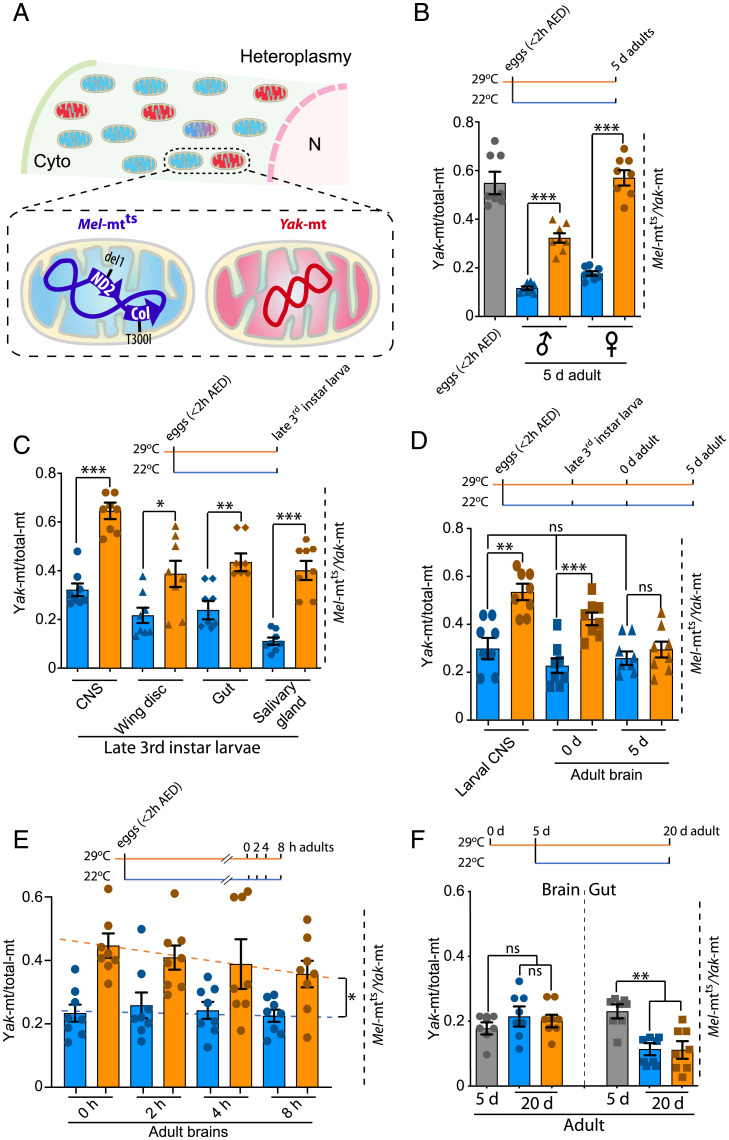
Quality control modulates the ratio of heteroplasmic mitochondrial genomes during development. (*A*) Heteroplasmy for schematized *Yak-*mt and *Mel-*mt^ts^ genomes was established by transferring cytoplasm of *D. yakuba* embryos into *D. melanogaster* embryos carrying the doubly mutant genome *mt:ND2^del^ + mt:Col^T300I^* (*Mel-*mt^t^*^s^*). (*B*) The proportion of *Yak-*mt (*Yak-*mt/total-mt) following development from egg to adult shows action of quality control. Eggs (2-h collection at 29 °C) were assayed (gray bar) or allowed to develop to 5 d after eclosion at 22 °C (blue) or 29 °C (amber). Adult females have a large contribution from oocyte mtDNA. (*C*) Quality control operates in multiple tissues with different effectiveness. The blue and amber bars (22 °C or 29 °C, respectively) show the proportion of *Yak-mt* in different tissues of late third instar larvae. (*D*) The impact of purifying selection declined with age in the CNS. (*E*) The decline in relative abundance of wild-type, indicated by the dashed regression lines (orange for 29°C and blue for 22°C), *Yak*-mt following eclosion was fast. (*F*) The signature of quality control is absent during maturation of adults. *Yak*-mt/total-mt ratios from gut and brain taken from 5-d (gray bars) or 20-d adults aged at 22 °C or 29 °C (blue and amber bars, respectively). Here and below, *P < 0.05; **P < 0.01; and ***P < 0.001 by one-way ANOVA/Tukey’s multiple comparison test. Data represent eight independent biological repeats, with each repeat being an average of ratios assessed in three samples of eggs or adults. For tissues, data represent tissues dissected from eight individuals. Error bars represent standard error. In *E*, slopes differ (*P < 0.05) by linear regression. Cyto, cytoplasm; AED, after egg deposition; ns, not significant.

A previously described heteroplasmic line of *Drosophila melanogaster* carries a wild-type mitochondrial genome (*Yak*-mt) from another species, *Drosophila yakuba*, and a *D. melanogaster* genome (*Mel*-mt^ts^) crippled by a temperature-sensitive mutation in cytochrome oxidase subunit 1 (*mt:CoI^T300I^*) [Fig. 1*A* and (25)]. The *Mel*-mt^ts^ genome has an intrinsic advantage in replication. At a permissive temperature, it gradually displaces the *Yak*-mt genome, resulting in the loss of the *D. yakuba* genome in a few generations (25). At 29 °C, a temperature at which the *CoI^T3000I^* mutant cannot support viability (26), purifying selection counters the replicative advantage of the mutant *Mel*-mt^ts^ genome, preventing it from taking over (25). qPCR gives a measure of the ratio *Yak*-mt/total-mt. The difference in this ratio between permissive and restrictive temperatures allows us to assess the impact of a functional disparity on competition between the two genomes and provides a measure of purifying selection.

Past work using this heteroplasmic line focused on changes in the relative abundance of the two genomes from one generation to the next and uncovered the action of purifying selection during oogenesis (18–21, 25, 27). It is notable that this selection depends on quality control, occurs by competition between mitochondria within the oocyte, and does not involve selection for organismal fitness (18, 21). Changes in the ratio of *Yak*-mt/total-mt during the lifetime of the fly suggested that maintenance of this ratio is dynamic (25). Here, we examined changes in the relative abundance of the two genomes in the soma. Shifts in the ratio of *Yak*-mt/total-mt provide a measure of the effectiveness of purifying selection during the life of the fly and a means of assessing whether purifying selection can be genetically or pharmacologically modified. We found evidence that purifying selection is active in the soma during growth and development. The influence of mutations in quality control genes suggests that somatic mechanisms of quality control overlap those operating in the germ line. However, the effectiveness of purifying selection declines with age and has no obvious impact in tested tissues beyond 5 d after eclosion of adult flies. Importantly, we found that quality control can be stimulated in the adult by genetic alterations or by feeding kinetin and that such measures forestall mutational accumulation and aging phenotypes in *Drosophila* and can reverse mutational accumulation in aged mice.

## Results

### Somatic Selection of Mitochondrial Genomes during Development and Aging.

To assess the action of selection in somatic tissues during development, we collected eggs from heteroplasmic flies at 29 °C, allowed development to young adulthood at either 22 °C or 29 °C, and measured the ratios of mitochondrial genomes in whole flies (Fig. 1*B*). For analysis of events in the soma, we focused on males because high ovarian mtDNA levels result in a large germ line influence in females (25). At 22 °C, the ratio of *Yak*-mtDNA to total-mtDNA declined dramatically from egg to adult, representing the outcome of competition when both genomes were functional. At 29 °C, *Yak*-mt/total-mt was maintained at a higher level than in flies raised at 22 °C. This difference reflects a response to functional disparity between the genomes and shows selection against the detrimental mutation. However, the *Yak*-mt/total-mt ratio in the 5-d adult males was lower than in freshly laid eggs, suggesting that this selection is weaker during growth to adulthood than it is in the female germ line (Fig. 1*B*).

To assess the influence of a functional disparity during earlier development, we examined larval tissues after development at 22 or 29 °C. Again, the *Yak*-mt/total ratio declined at 22 °C. The extent of the decline was different in the different tissues. Although the basis for this difference is not known, it might be attributed to tissue-specific influences on the replicative abilities of the two different genomes (3). However, regardless of the cause, the *Yak*-mt/total ratio at 22 °C serves as a control for change induced when *mt:CoI* is inactivated by increasing the temperature to 29 °C. Total mtDNA levels were similar at both temperatures (*SI Appendix*, Fig. S1), suggesting that copy number is not responsive to the difference in *mt:CoI* function. In contrast, the ratio of *Yak*-mt to total-mt was higher at 29 °C in all tissues examined (Fig. 1*C*). We conclude that some form of purifying selection contributes to mitochondrial genome quality in diverse larval tissues during development to the late larval stage.

Despite early purifying selection in larval stages and maintenance at 29 °C, the *Yak*-mt/total-mt ratio in the central nervous system (CNS)/brain declined during continued development (Fig. 1*D*). Indeed, 5 d after eclosion from the pupal case (5-d adult), the ratio was nearly at the level seen in the brains of flies that were raised the entire time at 22 °C (Fig. 1*D*). Measuring *Yak*-mt/total-mt every 2 h after eclosion revealed a rapid decline of the ratio (Fig. 1*E*). Thus, the transition to adulthood is accompanied by a rapid shift in the ratio of mitochondrial genomes that erases the benefits of earlier purifying selection in the brain. A decline in the effectiveness of purifying selection would precede its consequence on the *Yak*-mt/total-mt ratio. Thus, the rapidity of the posteclosion readjustment of *Yak*-mt/total-mt reveals a dynamic requirement for purifying selection.

Aging of adult flies from 5 to 20 d did not result in further decline of *Yak*-mt/total-mt in brain at either temperature (Fig. 1*F*). In contrast, the *Yak*-mt/total-mt fell further in the highly proliferative gut. However, it is notable that this decrease in the gut was the same at both temperatures. The temperature independence of *Yak*-mt/total-mt during aging indicates a lack of effective purifying selection against the detrimental mutation after 5 d of adulthood.

### Nuclear Genes Modulate Purifying Selection of Mitochondrial Genomes in the Soma.

**Fig. 2.**
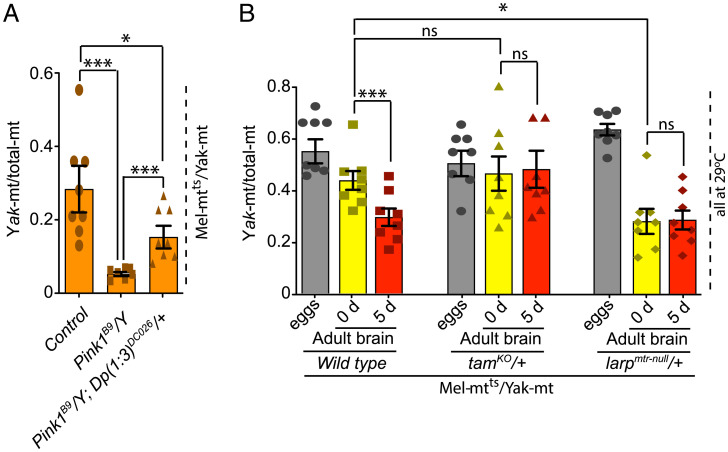
The nuclear genotype impacts the maintenance of mitochondrial genome quality in adult brains. (*A*) Loss-of-function of *Pink1* leads to decline in *Yak-*mt/total-mt, indicating that PINK1 helps sustain high levels of the functional genome. Data show genome ratios for 5-d-old flies of the indicated genotype raised at 29 °C. The *Pink1^B9^*/*Y* flies lack Pink1 function, while the *Pink1^B9^*/*Y*; *Dp(1:3)^DC026^*/*+* flies carry a rescuing autosomal duplication of the normally X chromosomal *PINK1* gene. The PINK1-deficient flies and rescued flies are sibs from the same cross and therefore have the same pool of maternally contributed mitochondrial genomes. (*B*) The influence of nuclear genotype on mitochondrial genome quality of adult brains. Bars show the ratio of *Yak-*mt/total-mt in eggs (gray) and in brains of newly eclosed (0 d: yellow) or 5-d-old (red) adults when the indicated genotypes are raised at 29 °C. Data reflect the consequences of zygotic dose-reduction of the tested gene. *P < 0.05; and ***P < 0.001 by one-way ANOVA/Tukey’s multiple comparisons test. Data represent eight independent biological repeats, with each repeat being an average of ratios assessed in three samples of eggs or adults. For tissues, data represent tissues dissected from eight individuals. Error bars represent standard error. ns, not significant.

Since PINK1 acts as a sensor in quality control pathways, we tested its influence on purifying selection in the soma. A loss-of-function mutation in the X chromosomal *Pink1* locus dramatically decreased *Yak*-mt/total-mt in 5-d adult male flies raised at 29 °C (Fig. 2*A*). This decline was significantly rescued by an autosomal copy of wild-type PINK1. We conclude that *Pink1* contributes to somatic purifying selection in flies.

To assess quality control events downstream of *Pink1*, we reduced the gene dose of *larp*, a mediator of *Pink1* quality control in the germ line (*SI Appendix*, Fig. S2) (18). Although changes in gene dose usually produce subtle or no phenotype, we reasoned that the decline in the *Yak*-mt/total-mt ratio in brains during the transition from pupa to adulthood might provide an especially sensitive point to monitor the impact of quality control in somatic tissues. Heterozygosity for *larp* compromised purifying selection so that the brains of newly eclosed flies had much reduced *Yak*-mt/total-mt (Fig. 2*B*). Additionally, a genome-wide screen showed that reduction in the dose of the gene *tamas* (*tam*), which encodes the mitochondrial DNA polymerase catalytic subunit (POLγA), enhanced elimination of mitochondrial genomes with deleterious mutations during oogenesis (27). When we reduced the dose of *tam* during growth and development, a higher *Yak*-mt/total-mt persisted in brains, indicating stronger purifying selection (Fig. 2*B* and *SI Appendix*, Figs. S3–S5). These findings show parallels between germ line and somatic purifying selection and implicate a common quality control pathway in the processes.

Adult *Yak*-mt/total-mt heteroplasmic flies are lethargic and short-lived (about 20 d) at 29 °C. Since heteroplasmic Pink1 mutants had reduced mitochondrial genome quality, we examined the influence of *Pink1* on the health of heteroplasmic flies. *Pink1* mutant flies that are otherwise wild type are viable at 22 and 29 °C. In contrast, while *Pink1* flies heteroplasmic for *Yak*-mt and *Mel*-mt^ts^ survive at 22 °C, they die during the first few days of adulthood at 29 °C. This finding shows that PINK1 contributes to survival of heteroplasmic flies when function of *Mel*-mt^ts^ is compromised. The death is likely the consequence of the much-reduced abundance of the functional mitochondrial genome (Fig. 2*A*).

**Fig. 3.**
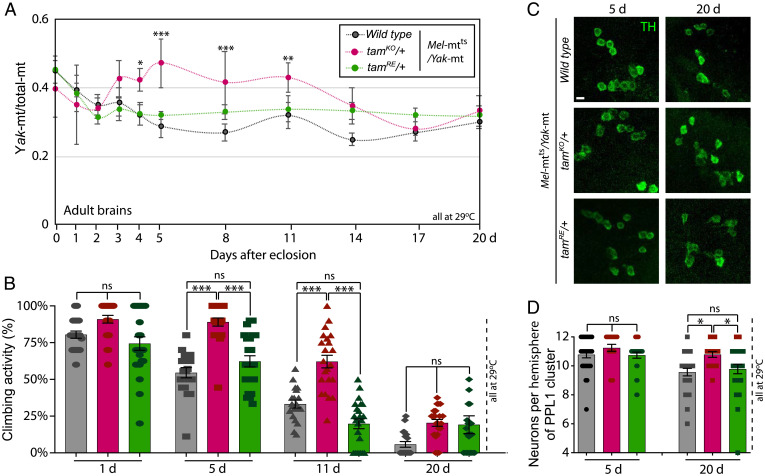
Genetically induced improvements in mitochondrial genome quality and vigor during aging. (*A*) A time course during adulthood shows the consequence of removing one of the two alleles of the gene encoding the mtDNA polymerase (*tam^KO^/+*) on *Yak-*mt/total-mt (red) compared to two controls (black is wild type, and green is a revertant *tam* allele). Flies were held at 29 °C and sampled at the indicated times. Data represent tissues dissected from eight individuals. (*B*) Removing one allele of mtDNA polymerase gene suppresses age-associated decline in climbing activity in heteroplasmic flies (*tam^KO^/+* in red, wild-type in gray, and revertant in green). Ten flies collected from an independent cross were grouped into a single task; 15∼25 tasks (150∼250 flies from 15 to 25 independent crosses) were tested for each genotype. (*C*) Example images of DA neurons (marked by anti-Tyrosine hydroxylase) in the PPL1 clusters of adult brains 20 d after eclosion, in heteroplasmic flies with the three nuclear genotypes indicated to the right of vertical bar. Data represent 20 to 30 brains collected from three independent experiments. (*D*) Quantification of DA neuron number in PPL1 clusters. *P < 0.05; **P < 0.01; and ***P < 0.001 by one-way ANOVA/Tukey’s multiple comparisons test. The error bars in A,B and D represent standard error. TH, tryosine hydroxylase; ns, not significant.

We then examined the influence of reducing the dose of *tam* during aging. When raised at 22 °C when both genomes are functional, there was no detected difference in *Yak*-mt/total-mt between control and *tam* heterozygous flies as they aged to 20 d (*SI Appendix*, Fig. S6). However, when raised at 29 °C, the heterozygous (*tam^KO^*/+) flies sustained a higher level of *Yak*-mt/total-mt for 14 d into adulthood (Fig. 3*A*). The relative impact of *tam* dose at the two temperatures is in accord with previous findings indicating that *tam* dose acts to enhance purifying selection (27).

To assess possible benefits of increased *Yak-*mt/total-mt, we examined the consequence of reducing the dose of *tam* at 29 °C. The *tam* heterozygotes showed more vigorous climbing at least until 11 d after eclosion (Fig. 3*B*). Neuronal cell loss has been associated with age-associated degenerative conditions. To test for such an association, we tested the PPL1 cluster of dopaminergic (DA) neurons for cell loss in control and in *tam^KO^* heterozygous flies. In the absence of heteroplasmy, normal flies exhibit an invariant number of PPL1 DA neurons throughout adult life (28). In contrast, DA neuron number dropped between 5 and 20 d of adulthood in the heteroplasmic flies. This decline was not detected in the *tam^KO^*/+ heteroplasmic line (Fig. 3*D*). The phenotypic benefits associated with maintenance of a higher proportion of functional genomes suggests that mitochondrial genome quality impacts age-associated degeneration.

### Kinetin Treatment Enhances Purifying Selection during Aging in Flies and Mice.

**Fig. 4.**
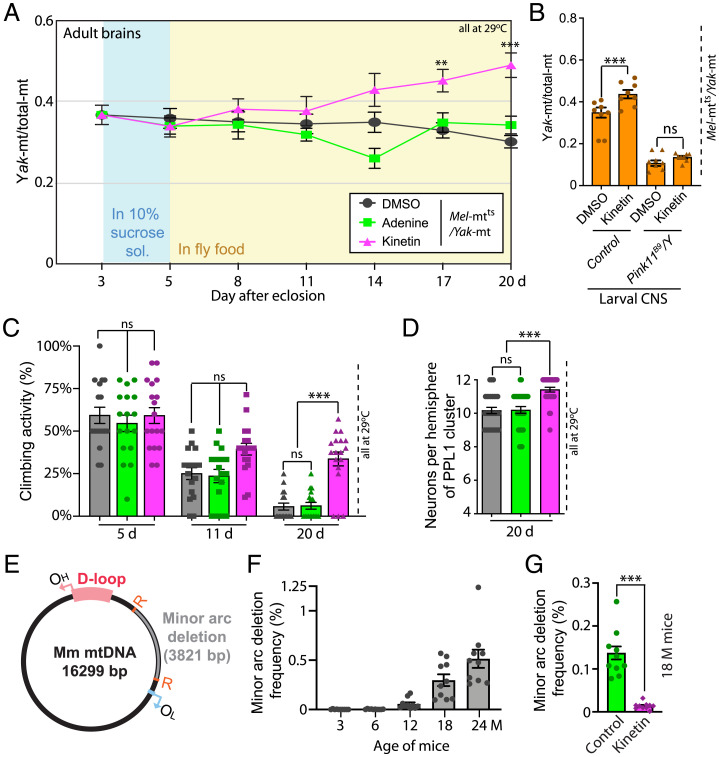
Pharmacological enhancement of PINK1 improves mitochondrial genome quality and vigor. (*A*) Kinetin treatment increases *Yak-*mt/total-mt in adulthood. Wild-type adult male heteroplasmic flies at 29 °C were fed (*Materials and Methods*) with solvent control (DMSO), 100 μM kinetin, or 100 μM adenine from day 3, and *Yak-*mt/total-mt was measured in brains at the times indicated. Data represent tissues dissected from eight individuals. (*B*) Kinetin-induced increase in *Yak-*mt/total-mt required *Pink1*. *Yak-*mt/total-mt increase in the CNS of control late third instar larvae (29 °C) that were fed kinetin, but not larvae lacking *Pink1*, *Pink1^B9^/Y*. Data represent tissues dissected from eight individuals. (*C*) Kinetin sustains climbing activity in heteroplasmic flies with age. Heteroplasmic males at 29 °C were fed with control (DMSO, gray), 100 μM adenine (green), or 100 μM kinetin (red) from day 3 and tested for climbing activity at the indicated times. Ten flies collected from an independent cross were grouped into a single task; 15∼25 tasks (150∼250 flies from 15 to 25 independent crosses) were tested for each genotype. (*D*) Kinetin treatment prevents neuronal loss in PPL1 clusters of heteroplasmic males. Quantification of neuron number of PPL1 clusters in each hemisphere in heteroplasmic male adult flies at 29 °C fed with DMSO, 100 μM adenine, or 100 μM kinetin from day 3 and assayed at day 20. Data represent 20 to 30 brains collected from three independent experiments. (*E*) Illustration of deletion (gray) located between two 9-bp repeat sequences (orange “R”) within minor arc. (*F*) Age dependency of deletion frequency in liver of C57BL6/J mice. Two samples from each of five mice were measured by ddPCR for each age group. (*G*) Kinetin effect on minor arc deletion levels in aged mouse liver. For 6 wk, 16.5-mo-old mice were fed chow supplemented with kinetin. This treatment reduced minor arc deletion levels 10-fold compared to control chow–fed mice of the same age. **P < 0.01; and ***P < 0.001 by one-way ANOVA/Tukey’s multiple comparisons test. The error bars represent standard error. sol., solution; OH and OL, origin for heavy and light strand replication, respectively; ns, not significant.

Encouraged by findings that genetic manipulations altered the balance of mitochondrial genomes, we wondered whether pharmaceutical activation of quality control might reverse mutational accumulation in aging animals. Kinetin is a modified form of adenine and a cytokine in plants (29). Kinetin and its ribosyl derivative have been shown to activate PINK1 in mammalian cells (30, 31). We tested the influence of kinetin during aging of adult flies. We fed 3-d-old adults raised either at 29 °C or 22 °C vehicle control (dimethyl sulfoxide [DMSO]), the natural purine (adenine, 100 µM), or kinetin (100 µM) and followed the ratio of mitochondrial genomes. While *Yak*-mt/total-mt was relatively stable in controls, in flies raised at 29 °C the ratio rose progressively in the kinetin-fed flies (Fig. 4*A*), while kinetin feeding had no effect at 22 °C (*SI Appendix*, Fig. S7), when we expect no discordance in the abilities of the two genomes to support electron transport. The effect of kinetin was dose dependent and was neutralized by adenine competitor (*SI Appendix*, Fig. S8). We concluded that kinetin treatment promotes purifying selection in our heteroplasmic line during aging.

Since *Pink1*-mutant heteroplasmic flies do not survive into later adulthood, to test whether the kinetin action depends on PINK1 function, we examined heteroplasmic larvae. Kinetin (50 µM) feeding increased *Yak*-mt/total-mt in the CNS of control heteroplasmic larvae at 29 °C. This action of kinetin was not detected in *Pink1* loss-of-function mutant larvae, consistent with the expectation that kinetin acts by stimulating PINK1 (Fig. 4*B*). Furthermore, while kinetin increased *Yak*-mt/total-mt in control heteroplasmic larvae at 29 °C, it had no effect on larvae at 22 °C (*SI Appendix*, Fig. S8*C*), paralleling results in adult flies (*SI Appendix*, Fig. S7). This temperature dependence suggests that the effect requires the functional distinction between the genomes as expected if kinetin acts by stimulating quality control.

We then tested whether increased *Yak*-mt/total-mt in older kinetin-treated flies is associated with improved well-being. Kinetin feeding sustained climbing activity (Fig. 4*C*) and maintained the DA neurons in PPL1 in aged heteroplasmic flies (Fig. 4*D*). Thus, kinetin treatment promotes purifying selection and mitigates the decline of vigor of heteroplasmic flies.

As in the fly model, competition between mutant and wild-type mitochondrial genomes ought to influence accumulation of mutations in mammals. If failing capabilities of purifying selection in aged mammals contribute to age-associated accumulation of mutations as in flies, perhaps kinetin treatment would suppress or reverse age-associated accumulation of a mitochondrial mutation in mice.

We used a sensitive digital PCR assay to measure age-associated accumulation of a naturally emerging deletion in the minor arc of mtDNA in liver of WT C57BL6/J mice (Fig. 4*F*). Note that because mutations are rare events, first emergence of the deletion will vary from mouse to mouse, leading to some quantitative variation. Nonetheless, mutational abundance rises dramatically in older mice, with a near-exponential response to age. We fed control or kinetin-containing food to 16.5-mo-old mice for 6 wk and assessed levels of the minor arc deletion in liver. Kinetin feeding reduced the level of the minor arc deletion to well below that of similarly aged mice fed control food (Fig. 4*G*). Moreover, kinetin reduced the mutation load below the level characteristic of the age at onset of kinetin feeding (*SI Appendix*, Fig. S9). This shows that a PINK1 activator reverses the age-associated increase of this mutant allele.

## Discussion

Random segregation of mitochondrial genomes can lead to stochastic variation in the relative abundance of co-resident genomes within the cells of an individual (6, 32), but selective forces often bias the outcome to drive directional changes (6, 19, 20, 22, 32–35). In the female germ line of *Drosophila*, quality control promotes biogenesis and proliferation of those mitochondria that maintain a useful membrane potential (18, 19, 36). This puts mitochondria compromised by deleterious mutations at a proliferative disadvantage, resulting in their elimination within a few generations. It has, however, been less clear how selective forces and quality control mechanisms influence the abundance of mutant genomes in the soma. The accumulation of mitochondrial mutations is important in the progression of heteroplasmic mitochondrial disease and impacts normal aging.

A genetic model for heteroplasmy in *Drosophila* provided a means of assessing selective forces that influence the abundance of a deleterious mutation. While the overall abundance of the genome with the deleterious mutation is influenced by a variety of selective forces, by using a temperature-sensitive mutation and assessing the results at two different temperatures, we can isolate the influence of the change in function of the *mt:CoI* gene product. We take the difference in abundance at the two temperatures as a measure of the impact of purifying selection. By introducing mutations altering quality control pathways, we can assess the role of the affected pathway in purifying selection. This allowed us to show that purifying selection operates in somatic tissues during development and growth (Fig. 1) and that a PINK1-dependent pathway contributes importantly to this purifying selection (Fig. 2*A*). Sensitivity to reduction in the dose of *larp* and enhancement by reduction in the dose of *tam* suggest that the mechanism operating in the soma is similar to that described for purifying selection during oogenesis (18), but there may be additional mechanisms contributing.

Selection can only influence the relative proportion of competing mitochondrial genomes if there is replication and/or turnover of the genomes. Thus, stability might contribute to the absence of observed purifying selection in the adult. However, this cannot explain periods of dynamic change in *Yak*-mt/total-mt. For example, the *Yak*-mt/total-mt ratio in the gut falls during adulthood, but falls the same amount at both temperatures (Fig. 1*F*). Additionally, we characterized a very rapid decline in *Yak*-mt/total-mt in the young adult following eclosion (Fig. 1*D* and *E*). Because this decline erased the benefits of earlier purifying selection, we interpret the change as the dynamic resetting of *Yak*-mt/total-mt ratio after a major reduction in the effectiveness of purifying selection.

We measured the impact of purifying selection rather than the process itself. Consequently, a rise in counteracting selection, rather than a loss in purifying selection, might be responsible for the reduction in the impact of purifying selection. The unexpected prevalence of deleterious mutations in adult mutator flies led to the suggestion that such mutations might be positively selected (23). Although we do not know how such a “destructive” selection would occur, if it exists, it would act in opposition to purifying selection and so reduce its impact. Accordingly, it is possible that a rise in destructive selection accounts for our recorded changes in adulthood.

Perhaps our most impactful finding is that purifying selection can be at least somewhat restored in later adulthood either genetically or pharmacologically (Figs. 3 and 4). In addition to improving mitochondrial genome quality, these treatments enhanced the climbing ability of aged heteroplasmic flies and suppressed loss of DA neurons (Figs. 3 and 4). This ability to reverse negative effects of heteroplasmy on the aging of flies suggests that such treatment might have the potential to reverse detrimental consequences of heteroplasmic mitochondrial diseases and perhaps suppress age-associated accumulation of mitochondrial mutations. Such possibilities are further bolstered by our finding that kinetin, the pharmacologic agent promoting purifying selection in adult flies, also worked in mice to suppress age-associated increase in a detrimental mtDNA mutation (Fig. 4).

Our findings raise questions about how the quality control mechanisms decline with age. A previous study, using a different model that assessed the levels of a deletion of mtDNA that was induced in adult *Drosophila* flight muscle, also found negligible levels of purifying selection (22). This study went on to demonstrate that a number of genetic alterations enhanced purifying selection. This led to the conclusion that “wild-type levels of key gene products are not set to maximize mtDNA quality control.” Specifically, this work implied that such processes as mitochondrial fusion and fission as well as the ability of mitochondrial adenosine 5′-triphosphate synthase to run in reverse are not optimized for quality control in the adult. Together with our findings, this report suggests a number of different pathways that are candidates for pharmacological modification that will benefit mtDNA quality during aging. While the findings also suggest that many different pathways might limit quality control in the adult, they do not explain why function is not optimized.

While it is puzzling why there is a lapse of purifying selection in the adult, we suggest that it may be fallout of the shifting priorities of evolution at different stages of life. Growth and development of metazoans occur in protected stable environments where organismal performance is not a factor, but maintenance of genome quality is key to the success of the future individual and to the production of future generations. Under these circumstances, evolution succeeds by prioritizing purifying selection. In contrast, the survival and reproductive potential of the adult depends on performance in a challenging and varied environment. At this life stage, evolution would select for elite performance and might optimize the energy-producing capacity of mitochondria by increasing the DNA content of weak mitochondria, even at the expense of triggering amplification of less functional genomes.

Regardless of the underlying reasons, we showed a decline in the impact of quality control in the adult and that a decline in vigor parallels reductions in the average quality of mitochondrial genomes during aging. These observations, together with substantial literature (6, 10–12, 18–22, 25, 32–35), suggest that the mitochondrial “genotype” of a metazoan is plastic and molded by selective forces acting within the organism. Importantly, we showed that mitochondrial genome quality can be genetically and pharmacologically modified during an organism’s lifetime to benefit well-being (Figs. 3 and 4). These findings bode well for development of therapies that modulate selective forces to enhance mitochondrial quality to benefit health span and lessen the impact of mitochondrial disease.

## Materials and Methods

### Fly Stocks.

The heteroplasmic fly (*Mel-*mt^ts^*/Yak-*mt) containing both a *D. melanogaster* mt genome with two mutant alleles (*mt:ND2^del1^* + *mt:CoI^T300I^*) and a *D. yakuba* mitochondrial genome was previously described (25). We used a derived stock of the original heteroplasmic line (25) that carries a higher proportion of *Yak-mt*. *Mel-*mt^ts^*/Yak-*mt females were backcrossed to males of a laboratory version of Canton S for 10 generations to homogenize the nuclear background (the laboratory stock was itself created by ingression of a Canton S background into *w1118*). The *tam^RE^* and *tam^KO^* alleles are congenic transgenic flies with a restored wild-type allele of *tam* (control) and a knockout allele, respectively [gifts from Dr. Anna Wredenberg, Karolinska Institutet, Stockholm, Sweden(37)]. *larp^mtr-null^* originated from Dr. David M. Glover, University of Cambridge, Cambridge, UK and was a gift from Dr. Hong Xu, NIH, Bethesda, MD (17). *tam^3^*, *tam^4^*, *PINK1^RV^*, *PINK1^B9^*, and *Dp(1:3) ^DC026^* were obtained from the Bloomington *Drosophila* Stock Center. The stocks were cultured at 22 and 29 °C on standard fly medium.

### Mice.

Male C57BL/6J mice were obtained from The Jackson Laboratory, housed in a specific pathogen-free facility with a standard 12-h light/dark cycle at the University of California, San Francisco, and given food and water ad libitum. Experiments were conducted in accordance with institutional guidelines approved by the University of California, San Francisco Institutional Animal Care and Use Committee.

### DNA Isolation.

Total DNA of adult flies was extracted as follows. Three adult flies were pooled and mechanically homogenized with a plastic pestle in 105 μL homogenization buffer (100 mM Tris⋅HCl [pH 8.8], 0.5 mM ethylenediaminetetraacetic acid (EDTA), and 1% sodium dodecyl sulfate [SDS]). The homogenate was incubated at 65° for 30 min, followed by addition of 15 μL potassium acetate (8 M) and incubation on ice (30 min) to precipitate protein and SDS. Subsequently, the homogenate was centrifuged at 13,000 rpm for 15 min at 4 °C. DNA was recovered from the supernatant by adding 0.5 volume of isopropanol and centrifuging at 20,000 × *g* for 5 min at room temperature. The resultant pellet was washed with 70% ethanol and suspended in 20 μL double distilled water (ddH_2_O). DNA was extracted from tissue samples as follows. A single dissected fly tissue (brain, wing disk, or gut) was placed on a clean cover slide, mixed with 10 μL lysis buffer (10 mM Tris⋅HCl [pH 8.8], 1 mM EDTA, 25 mM NaCl, 1% SDS, and 8 U/mL Proteinase K, NEB) for 3 min at room temperature. The tissue/buffer mixture was transferred to a 0.2-mL tube and incubated at 37°C for 30 min and heat inactivated by 95 °C for 5 min.

Mice were anesthetized by intraperitoneal injection of 0.3 cc 17.5 mg/mL Ketamine and 10 mg/mL Xylazine mixture in saline and perfused with 15 to 20 mL sterile phosphate-buffered saline (PBS) through the heart left ventricle using the Surflo winged infusion set (Terumo Corporation, SV-21BLK) attached to a 30-mL syringe. After perfusion, livers were collected, immediately frozen on dry ice, and stored at −80 °C until DNA isolation. Two small liver fragments (∼1 mm^3^) per animal were analyzed. Fragments were lysed in 90 μL lysis solution (50 mM Tris⋅HCl [pH 8.0], 10 mM EDTA [pH 8.0], 0.5% SDS, and 2 mg/mL proteinase K [Promega, v3021]) at 37 °C overnight followed by addition of 10 μL of 3M potassium acetate and incubation at room temperature for 1 h. Next, lysates were centrifuged at 21,000 × *g* for 10 min, and supernatants were transferred to silica membrane spin columns (Macherey-Nagel) and centrifuged at 11,000 × *g* for 1 min. Flow-throughs were heat inactivated at 70 °C for 30 min and cleaned up using homemade magnetic beads [1:1 ratio (38)]. After cleanup, DNA was eluted in I mM Tris⋅HCl [pH 8.0], 0.1 mM EDTA and stored at 4 °C.

### qPCR Analyses.

For all qPCR assays, SYBR Select Master Mix (Applied Biosystems, 4470908) was used in 20-μL reactions with 400 nM of each primer. To measure the total mtDNA copy number of heteroplasmic flies, qPCR of a 128-bp *mt:IrRNA* region present in both mtDNA genotypes was performed (primer AACCAACCTGGATTACACCG and TGCGACCTCGATGTTGGATT) and normalized to nuclear genome copy number of the *αTub84D* gene (primer ATGCGCGAATGTATCTCTATCC and AGGTGTTAAACGAGTCATCACC). To measure copy number of *D. yakuba* genomes, qPCR of the 71-bp *D. yakuba mt:COXI* region was performed (primer AGTTGAAAACGGAGCTGGTACA and CTCCACCATGAGCGATACCTG). The efficiency and specificity of *D. yakuba mt:COXI* and total *mt:IrRNA* primer sets in qPCR reaction were normalized each time by analyzing the ratio between the two primer sets in DNA samples from a *D. melanogaster* wild-type stock of Canton S. The background for the *Yak-*mt–specific reaction was also assessed in every qPCR experiment and was always less than 0.005% of total-mt in DNA samples from Canton S. qPCR was performed with the following reaction conditions: 95 °C for 3 min, followed by 40 cycles at 95 °C for 5 s and 62.5 °C for 15 s. For each 20-μL qPCR reaction, 0.375% of an adult fly’s or 0.667% of a tissue’s total genomic DNA was used as a template. The percentage of *D. yakuba* mtDNA was calculated by dividing *D. yakuba* mtDNA copy number by the total mtDNA copy number. The threshold cycle (Ct) values used ranged from 18 to 30.

### ddPCR Analyses.

The droplet digital polymerase chain reaction (ddPCR) analysis was performed on a QX100 system (Bio-Rad). Two assays designed to detect minor arc deletion (primer LD_F CCAGAAGATTTCATGACC, primer LD_R CTTCTAGGTAATTAGTTGGG, probe LD AACTAATCCTAGCCCTAGCC) and common sequence for both deleted and nondeleted mitochondrial genomes (primer LD_common_F TTAGGATATACTAGTCCGC, primer LD_common_R GAAGTCTTACAGTCCTTATC, probe LD_common AGCCTTCAAAGCCCTAAGA) were used simultaneously. Template DNA concentration was adjusted to be below 3,500 mitochondrial genome copies per microliter of ddPCR reaction mixture. The reaction mixture (total volume 22  μL) contained 11  μL of 2× ddPCR Super Mix for Probes (Bio-Rad, 1863024), 1.1 μL of four primers mixture, 18 μM each, 1.1 μL of 5  μM probe LD_common labeled with HEX (IDT), 1.1 μL of 5 μM probe LD labeled with FAM (IDT), 0.5  μL of Alu1 restriction enzyme (NEB, R0137L), 2.2 μL of ddH_2_O, and 5 μL of template DNA. Then, 20 μL of the reaction mixture and 70 μL of oil (Bio-Rad, 1863005) were loaded on a DG8 cartridge (Bio-Rad, 1864007) for droplet generation on QX100 Droplet Generator (Bio-Rad), and 40 μL of droplet emulsion were transferred to 96-well plate (Bio-Rad, 12001925) and sealed with a pierceable foil (Bio-Rad, 1814040) using PX1 PCR plate sealer (Bio-Rad). The optimized PCR thermal cycling was conducted on a conventional PCR machine (Bio-Rad, C1000 Touch) using the following conditions: 10-min activation period at 95 °C, followed by 40 cycles of denaturation at 94 °C for 30 s, ramp rate 2 °C, and combined annealing-extension at 54 °C for 1 min, ramp rate 2 °C, and one cycle of 98 °C for 10 min. After thermocycling, samples were analyzed on the QX100 Droplet Reader (Bio-Rad). Results were analyzed with QuantaSoft Analysis Pro v.1.0.596 software (Bio-Rad).

### Behavior Assay.

A number of independent crosses, 15, 22, or 25, were carried out to produce flies of each genotype: wild-type, tam^KO^/+, or tam^RE^/+, respectively. Ten male flies were collected from each cross to form 62 independent cohorts. Each cohort was challenged in a climbing test done at days 1, 5, 11, and 20. In each test, climbing ability was defined as the ability of the adult fly to climb 5 cm within 10 s. If the fly could accomplish the task (≤10 s), it was given a score of 1; otherwise, it was given a score of 0 (≥10 s). All surviving flies were scored, and the % success of the surviving flies was tabulated and plotted. In total, 248 independent tests were conducted.

### Immunocytochemistry and Confocal Microscopy.

Adult brains were dissected in PBT (0.3% Tween 20 in PBS) and incubated with fixation solution (4% formaldehyde in PBT) for 20 min, followed by blocking for 1 h with 1% bovine serum albumin in PBT. Samples were immunostained with rabbit anti–Tyrosine hydroxylase (AB152; EMD Millipore Corporation) at 1:200 and Alexa 488–conjugated anti-rat (ab150165; AbCam). Samples were imaged with a 20X dipping 2-mm working distance objective on a Zeiss Confocal Laser-Scanning Microscope 780 with identical imaging parameters among different genotypes in a blind fashion. Images were processed with Photoshop CS4 using only linear adjustment of contrast.

### Kinetin Treatment in Fly.

For larval treatment, 0- to 6-h eggs were collected and grown on regular fly food (10 mL) containing DMSO (0.1%) with or without 50 μM kinetin. Six hours after egg collection, a 200 μL solution of 5% DMSO without or with 5 mM kinetin was applied on the top of the fly food for control and experimental larvae, respectively. Tissues were dissected from late third instar larvae.

For adult treatment, 3-d-old male adults were collected and first kept for 48 h in vials with a cotton ball moistened with 2 mL of a 10% sucrose, 2% DMSO solution (control) or the same supplemented with 100 μM kinetin, 100 μM adenine, or both 100 μM kinetin and 100 μM adenine. Then, the flies were transferred to vials prepared as described below, and passaged to similar fresh vials every 2 d until dissection. Recipient vials contained 10 mL of regular fly food with 0.1% DMSO (control) or the same supplemented with 100 μM kinetin, 100 μM adenine, or both 100 μM kinetin and 100 μM adenine. Prior to use, 200 μL of 5% DMSO (control), or the same supplemented with 5 mM kinetin, 5 mM adenine, or both 5 mM kinetin and 5 mM adenine was applied on the surface of the fly food and air-dried.

### Kinetin Chow.

Kinetin (Sigma-Aldrich) was delivered orally to mice in their chow following published reports (39). Rodent chow (Purina, 5053) was formulated by Research Diets to contain 3.50 g kinetin/kg chow for mice. These amounts of kinetin were well tolerated during trials according to a previous report (39). Chow was stored at −80 °C. Fresh chow was provided at least every 4 d. The delivery of chow was not blinded with respect to drug treatment. The 16.5-mo-old mice were fed with kinetin or control chow for 6 wk before sample collections at 18 mo of age. Body weights and chow intake were monitored at least twice weekly.

## Supplementary Material

Supplementary File

## Data Availability

All study data are included in the article and/or *SI Appendix*.

## References

[1] N. W. Soong, D. R. Hinton, G. Cortopassi, N. Arnheim, Mosaicism for a specific somatic mitochondrial DNA mutation in adult human brain. Nat. Genet. 2, 318–323 (1992). 1303287 10.1038/ng1292-318

[2] Y. Michikawa, F. Mazzucchelli, N. Bresolin, G. Scarlato, G. Attardi, Aging-dependent large accumulation of point mutations in the human mtDNA control region for replication. Science 286, 774–779 (1999). 10531063 10.1126/science.286.5440.774

[3] D. C. Samuels , Recurrent tissue-specific mtDNA mutations are common in humans. PLoS Genet. 9, e1003929 (2013). 24244193 10.1371/journal.pgen.1003929 PMC3820769

[4] H. L. Baines , Similar patterns of clonally expanded somatic mtDNA mutations in the colon of heterozygous mtDNA mutator mice and ageing humans. Mech. Ageing Dev. 139, 22–30 (2014). 24915468 10.1016/j.mad.2014.06.003 PMC4141908

[5] A. Trifunovic , Premature ageing in mice expressing defective mitochondrial DNA polymerase. Nature 429, 417–423 (2004). 15164064 10.1038/nature02517

[6] J. P. Jenuth, A. C. Peterson, E. A. Shoubridge, Tissue-specific selection for different mtDNA genotypes in heteroplasmic mice. Nat. Genet. 16, 93–95 (1997). 9140402 10.1038/ng0597-93

[7] J. B. Stewart, C. Freyer, J. L. Elson, N. G. Larsson, Purifying selection of mtDNA and its implications for understanding evolution and mitochondrial disease. Nat. Rev. Genet. 9, 657–662 (2008). 18695671 10.1038/nrg2396

[8] T. Tatsuta, T. Langer, Quality control of mitochondria: Protection against neurodegeneration and ageing. EMBO J. 27, 306–314 (2008). 18216873 10.1038/sj.emboj.7601972 PMC2234350

[9] A. J. Whitworth, L. J. Pallanck, The PINK1/Parkin pathway: A mitochondrial quality control system? J. Bioenerg. Biomembr. 41, 499–503 (2009). 19967438 10.1007/s10863-009-9253-3

[10] C. Vives-Bauza , PINK1-dependent recruitment of Parkin to mitochondria in mitophagy. Proc. Natl. Acad. Sci. U.S.A. 107, 378–383 (2010). 19966284 10.1073/pnas.0911187107 PMC2806779

[11] S. Geisler , PINK1/Parkin-mediated mitophagy is dependent on VDAC1 and p62/SQSTM1. Nat. Cell Biol. 12, 119–131 (2010). 20098416 10.1038/ncb2012

[12] D. P. Narendra , PINK1 is selectively stabilized on impaired mitochondria to activate Parkin. PLoS Biol. 8, e1000298 (2010). 20126261 10.1371/journal.pbio.1000298 PMC2811155

[13] A. C. Poole , The PINK1/Parkin pathway regulates mitochondrial morphology. Proc. Natl. Acad. Sci. U.S.A. 105, 1638–1643 (2008). 18230723 10.1073/pnas.0709336105 PMC2234197

[14] Y. Yang , Pink1 regulates mitochondrial dynamics through interaction with the fission/fusion machinery. Proc. Natl. Acad. Sci. U.S.A. 105, 7070–7075 (2008). 18443288 10.1073/pnas.0711845105 PMC2383971

[15] H. Deng, M. W. Dodson, H. Huang, M. Guo, The Parkinson’s disease genes *pink1* and *parkin* promote mitochondrial fission and/or inhibit fusion in *Drosophila*. Proc. Natl. Acad. Sci. U.S.A. 105, 14503–14508 (2008). 18799731 10.1073/pnas.0803998105 PMC2567186

[16] A. M. Pickrell, R. J. Youle, The roles of PINK1, parkin, and mitochondrial fidelity in Parkinson’s disease. Neuron 85, 257–273 (2015). 25611507 10.1016/j.neuron.2014.12.007 PMC4764997

[17] Y. Zhang, Y. Chen, M. Gucek, H. Xu, The mitochondrial outer membrane protein MDI promotes local protein synthesis and mtDNA replication. EMBO J. 35, 1045–1057 (2016). 27053724 10.15252/embj.201592994 PMC4868955

[18] Y. Zhang , PINK1 inhibits local protein synthesis to limit transmission of deleterious mitochondrial DNA mutations. Mol. Cell 73, 1127–1137.e5 (2019). 30772175 10.1016/j.molcel.2019.01.013 PMC7485087

[19] H. Ma, H. Xu, P. H. O’Farrell, Transmission of mitochondrial mutations and action of purifying selection in *Drosophila melanogaster*. Nat. Genet. 46, 393–397 (2014). 24614071 10.1038/ng.2919 PMC4091738

[20] J. H. Hill, Z. Chen, H. Xu, Selective propagation of functional mitochondrial DNA during oogenesis restricts the transmission of a deleterious mitochondrial variant. Nat. Genet. 46, 389–392 (2014). 24614072 10.1038/ng.2920 PMC3976679

[21] T. Lieber, S. P. Jeedigunta, J. M. Palozzi, R. Lehmann, T. R. Hurd, Mitochondrial fragmentation drives selective removal of deleterious mtDNA in the germline. Nature 570, 380–384 (2019). 31092924 10.1038/s41586-019-1213-4 PMC6614061

[22] N. P. Kandul, T. Zhang, B. A. Hay, M. Guo, Selective removal of deletion-bearing mitochondrial DNA in heteroplasmic *Drosophila*. Nat. Commun. 7, 13100 (2016). 27841259 10.1038/ncomms13100 PMC5114534

[23] C. L. Samstag , Deleterious mitochondrial DNA point mutations are overrepresented in *Drosophila* expressing a proofreading-defective DNA polymerase γ. PLoS Genet. 14, e1007805 (2018). 30452458 10.1371/journal.pgen.1007805 PMC6289449

[24] M. A. Walker , Purifying selection against pathogenic mitochondrial DNA in human T cells. N. Engl. J. Med. 383, 1556–1563 (2020). 32786181 10.1056/NEJMoa2001265 PMC7593775

[25] H. Ma, P. H. O’Farrell, Selfish drive can trump function when animal mitochondrial genomes compete. Nat. Genet. 48, 798–802 (2016). 27270106 10.1038/ng.3587 PMC4925267

[26] Z. Chen , Genetic mosaic analysis of a deleterious mitochondrial DNA mutation in *Drosophila* reveals novel aspects of mitochondrial regulation and function. Mol. Biol. Cell 26, 674–684 (2015). 25501370 10.1091/mbc.E14-11-1513 PMC4325838

[27] A. C. Chiang, E. McCartney, P. H. O’Farrell, H. Ma, A genome-wide screen reveals that reducing mitochondrial DNA polymerase can promote elimination of deleterious mitochondrial mutations. Curr. Biol. 29, 4330–4336.e3 (2019). 31786061 10.1016/j.cub.2019.10.060 PMC6926476

[28] K. E. White, D. M. Humphrey, F. Hirth, The dopaminergic system in the aging brain of *Drosophila*. Front. Neurosci. 4, 205 (2010). 21165178 10.3389/fnins.2010.00205 PMC3002484

[29] C. O. Miller, F. Skoog, M. H. Von Saltza, F. M. Strong, Kinetin, a cell division factor from deoxyribonucleic acid. J. Am. Chem. Soc. 77, 1392 (1955).

[30] N. T. Hertz , A neo-substrate that amplifies catalytic activity of Parkinson’s-disease-related kinase PINK1. Cell 154, 737–747 (2013). 23953109 10.1016/j.cell.2013.07.030 PMC3950538

[31] L. Osgerby , Kinetin riboside and its ProTides activate the Parkinson’s disease associated PTEN-induced putative kinase 1 (PINK1) independent of mitochondrial depolarization. J. Med. Chem. 60, 3518–3524 (2017). 28323427 10.1021/acs.jmedchem.6b01897 PMC5410652

[32] D. C. Samuels, P. Wonnapinij, L. M. Cree, P. F. Chinnery, Reassessing evidence for a postnatal mitochondrial genetic bottleneck. Nat. Genet. 42, 471–472, author reply 472–473 (2010). 20502486 10.1038/ng0610-471

[33] Y. S. Ju ; ICGC Breast Cancer Group; ICGC Chronic Myeloid Disorders Group; ICGC Prostate Cancer Group, Origins and functional consequences of somatic mitochondrial DNA mutations in human cancer. eLife 3, e02935 (2014). 10.7554/eLife.02935 PMC4371858 25271376

[34] J. N. Jasmin, C. Zeyl, Rapid evolution of cheating mitochondrial genomes in small yeast populations. Evolution 68, 269–275 (2014). 24372606 10.1111/evo.12228

[35] E. Bastiaans , Regular bottlenecks and restrictions to somatic fusion prevent the accumulation of mitochondrial defects in *Neurospora*. Philos. Trans. R. Soc. Lond. B Biol. Sci. 369, 20130448 (2014). 24864316 10.1098/rstb.2013.0448 PMC4032522

[36] D. Narendra, A. Tanaka, D. F. Suen, R. J. Youle, Parkin is recruited selectively to impaired mitochondria and promotes their autophagy. J. Cell Biol. 183, 795–803 (2008). 19029340 10.1083/jcb.200809125 PMC2592826

[37] A. Bratic , Complementation between polymerase- and exonuclease-deficient mitochondrial DNA polymerase mutants in genomically engineered flies. Nat. Commun. 6, 8808 (2015). 26554610 10.1038/ncomms9808 PMC4773887

[38] N. Rohland, D. Reich, Cost-effective, high-throughput DNA sequencing libraries for multiplexed target capture. Genome Res. 22, 939–946 (2012). 22267522 10.1101/gr.128124.111 PMC3337438

[39] A. L. Orr , Long-term oral kinetin does not protect against α-synuclein-induced neurodegeneration in rodent models of Parkinson’s disease. Neurochem. Int. 109, 106–116 (2017). 28434973 10.1016/j.neuint.2017.04.006 PMC5641232

