# Supplementary material for: Mitigation of age-dependent accumulation of defective mitochondrial genomes
Source: Proc Natl Acad Sci U S A. 2022 Jul 26;119(31):e2119009119. doi: 10.1073/pnas.2119009119 (PMC9351377; doi:10.1073/pnas.2119009119)
Supplement: Supplementary File [file pnas.2119009119.sapp.pdf]

Supplementary Information for

Mitigation of age-dependent accumulation of defective mitochondrial genomes.

Pei-I Tsai, Ekaterina Korotkevich & Patrick H. O'Farrell*

Department of Biochemistry and Biophysics, University of California, San Francisco, San Francisco, California, USA.

*Correspondence should be addressed to P.H.O (ofarrell@cgl.ucsf.edu)

Figures S1 to S9

Figure S1.

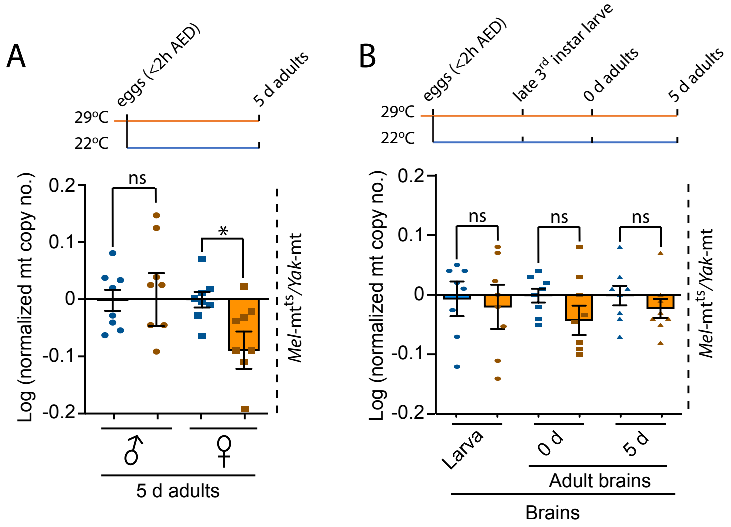

Fig. S1. Activating quality control by temperature inactivation of *mt:CoI*^{ts} does not alter total mt copy number. The log of a ratio of qPCR measurements (here and below) represent up and down changes in mtDNA copy number symmetrically around zero. The left sample acts as the control (normalizing) value. **(*A*)** In contrast to the influence of temperature on mt genome quality (Fig. 1), an increase in the temperature of development from 22 to 29°C did not alter the total copy number in males. The decrease in copy number in females at 29°C is likely due to reduced ovarian production of oocytes at the high temperature. Three adults were grouped as one biological sample and 8 biological repeats were measured for each condition. **(*B*)** Temperature was not associated with significant change in mt copy number in the brain. In the three pairs of bars, the 29°C data is normalized to the 22°C data for the same stage to show an absence of temperature effect. For each time and condition in **(*B*)** each data point is a single whole brain from one individual and 8 data points were collected at each time and stage. * = $p < 0.05$ by one-way ANOVA/Tukey's multiple comparison test. Error bars represent SE.

Figure S2.

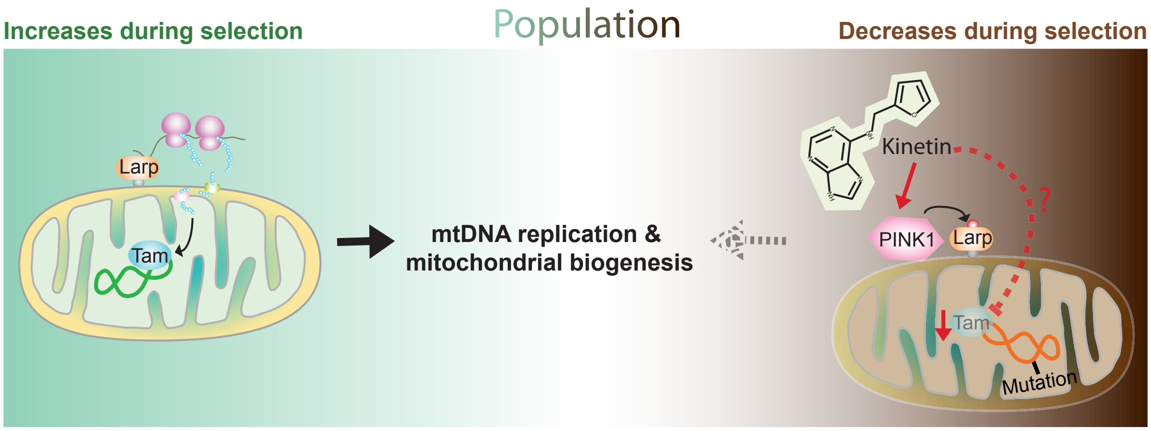

Fig. S2. Model for Kinetin/PINK1 regulation through Tamas in selective amplification of mitochondrial genomes in somatic tissues. Mitochondrial biogenesis is driven largely by translation of nuclear encoded mRNAs whose protein products are transported into mitochondria. The targeting of relevant mRNAs to mitochondria and local translation facilitate multiplication of mitochondria. Larp-based recruitment of appropriate mRNAs to the mitochondria underlies this local translation. The mRNA for the mitochondrial DNA polymerase, POLG (Tam in *Drosophila*), is among the mRNAs recruited to mitochondria. As described by Zhang et al., 2019, the system discriminates against unhealthy mitochondria, which accumulate the serine/threonine kinase PINK1 on the surface where it phosphorylates Larp, inhibiting its ability to recruit mRNAs for local translation. The result is selective mitochondrial biogenesis that enhances proliferation of healthy mitochondria giving them a selective advantage. While the system impacts the local translation of many mitochondrial proteins, in situations in which Tam is limiting, its selective translation might directly promote selective replication of genomes associated with healthy mitochondria (dashed red line). If other mitochondrial proteins are limiting, coupling of mtDNA replication to biogenesis ought to indirectly produce the same selective pressure. In a dynamic population with turnover, this selective biogenesis will progressively replace mutant mitochondria with healthy mitochondria, a form of intracellular purifying selection. However, when this purifying selection weakens with age, proliferation of defective mitochondrial genomes will no longer be constrained. By treating with Kinetin to re-activate PINK1 in elderly animals, we enhance quality control to restore purifying selection, improve the mean quality of the population of mitochondrial genomes, and improve vigor.

Figure S3.

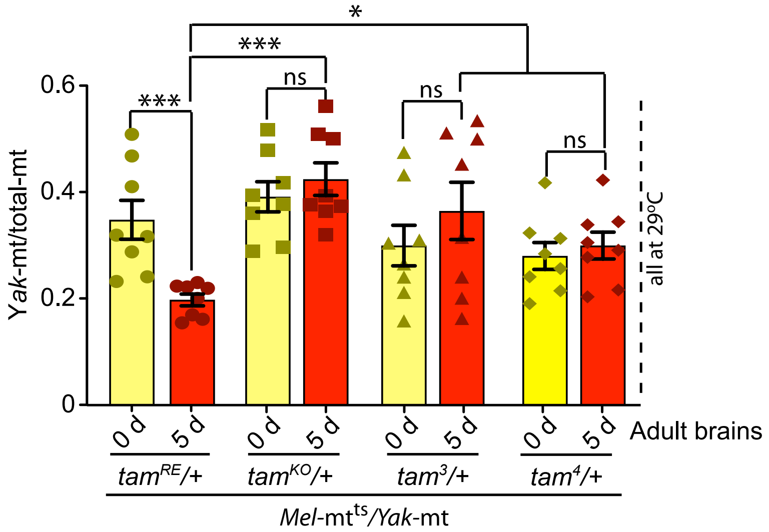

Fig. S3. Different alleles of *tam* all reduce the age-associated drop in *Yak*-mit/total-mt. Different mutant alleles of *tam* (*tam*^{KO}, *tam*^{3} and *tam*^{4}) all cause persistence of mitochondrial genome quality during early adulthood when heterozygous. *tam*^{RE} is a revertant allele congenic with *tam*^{KO} that is used as a control. Bars show the average ratio of *Yak*-mt to total-mt in brains dissected from newly eclosed (yellow) or 5-day old (red) adults of the stated genotypes that were raised at 29°C. For each stage, time and genotype, 8 brains were collected from individuals for mtDNA analyses. * = $p < 0.05$, ** = $p < 0.01$, and *** = $p < 0.001$ by one-way ANOVA/Tukey's multiple comparison test. Error bars represent SE.

Figure S4

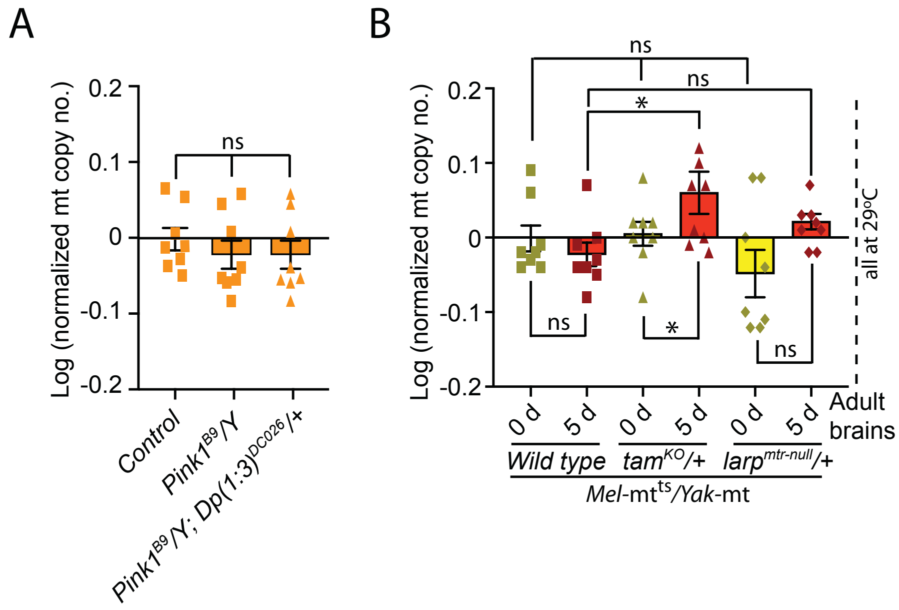

Fig. S4. Dosage of quality control genes have minimal effects on mtDNA copy number in young adults. (***A***) In contrast to the decline on mt genome quality, (Fig. 2), loss-of-function of *Pink1* did not alter the total copy number. (***B***) We also measured total mt copy number at eclosion (0 d) and at 5 days of adulthood (5 d) in heteroplasmic strains with a nuclear genome that was wild type or heterozygous for either *tam* or *larp*, as indicated. The flies were maintained at 29°C. Compared to newly eclosed wild type, small shifts were observed in the copy number of the heterozygous mutant lines, but only the slightly increased level in tam^{KO}/+ scored as modestly significant. For each time and genotype, we tested DNA from 8 whole brains that were collected from different individuals. * = $p < 0.05$ by one-way ANOVA/Tukey's multiple comparison test. Error bars represent SE.

Figure S5

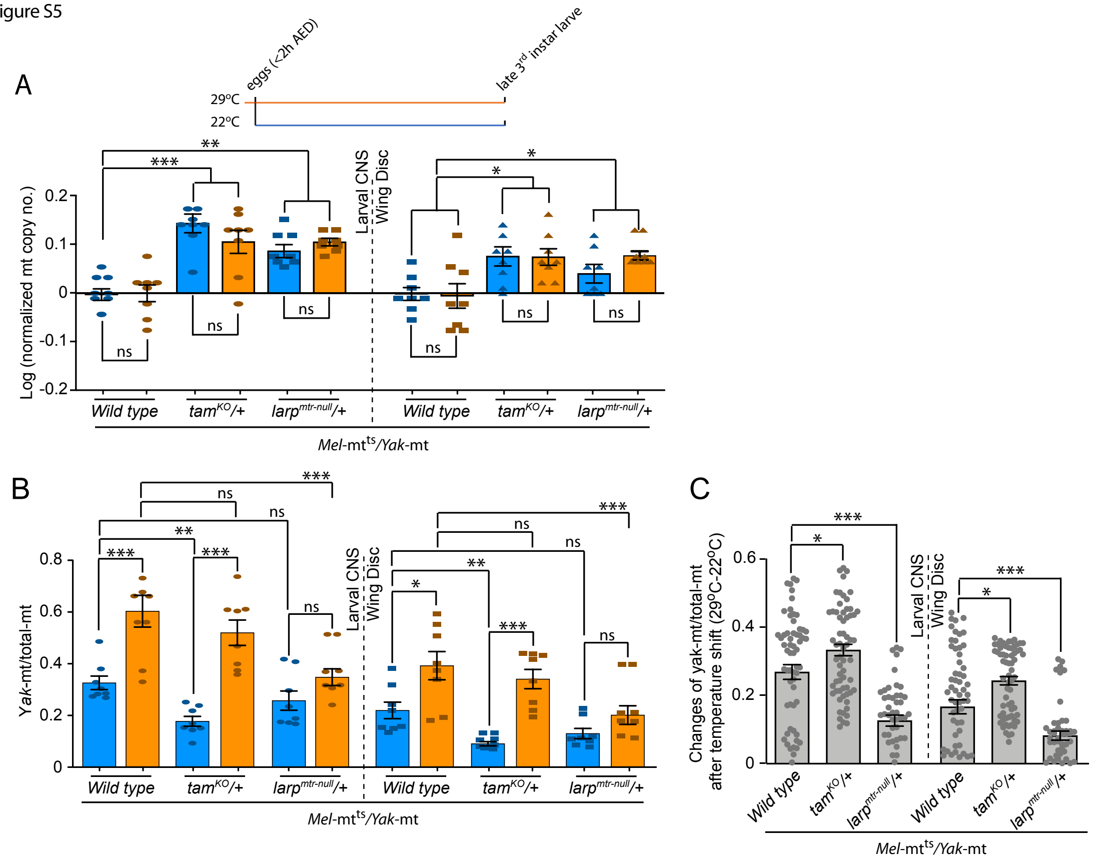

Fig. S5. Nuclear genotype has multiple influences on larval heteroplasmy. (***A***) Heterozygosity for *tam* or *larp* increased the copy number of total mtDNA irrespective of temperature. Shown for two larval tissues, wing disc and CNS from heteroplasmic stains (*Mel-mt*^{ts}*/Yak-mt*) with indicated nuclear genotypes raised at 22°C (blue) or at 29°C (amber) and normalized to wild-type 22°C. This suggests that the dose of these genes have a mild effect on copy-number control that is independent of the discordance in the functionality of the two genomes. (***B***) Nuclear genotypes have a complex influence on the ratio of *Yak*-mt/total-mt in larval tissues. The ratio of mt genomes was measured in two larval tissues (CNS and wing disc), at two temperatures (22°C and 29°C) and for the three nuclear backgrounds, wild type and both heterozygous mutants (*tam*^{KO}/+ and *larp*$^{mtr\text{-}null}$/+). Higher temperature increased the *Yak*-mt/total mt ratio (improved mt quality) in all nuclear genotypes but the increase in the *larp* heterozygote fell below significance, and heterozygosity for *tam* decreased the *Yak*-mt/total-mt at 22°C. (***C***) The two genes, *tam* and *larp*, have opposite but quantitatively small effects on quality control in these larval tissues. The influence of nuclear genotype on quality control is not easily seen in panel ***B*** because of the complicating influence of the nuclear genotype on the ratio of the two mitochondrial genomes at 22°C where both genomes are functional. To isolate the effect on quality control, we plotted the difference between 22°C and 29°C for each genotype, plotting the level at 29°C minus the level at 22°C. The results show that heterozygosity for *tam* modestly enhanced quality control, while heterozygosity for *larp* substantially compromised it, behaviors consistent with the effects of these mutations seen at the adult stage. While ***B*** plots 8 data points, in ***C***, each of 8 samples at one temperature was compared to every sample (8) measured at the other temperature to give 64 values that are plotted. * = $p < 0.05$, ** = $p < 0.01$, and *** = $p < 0.001$ by one-way ANOVA/Tukey's multiple comparison test. Error bars represent SE**.**

Figure S6

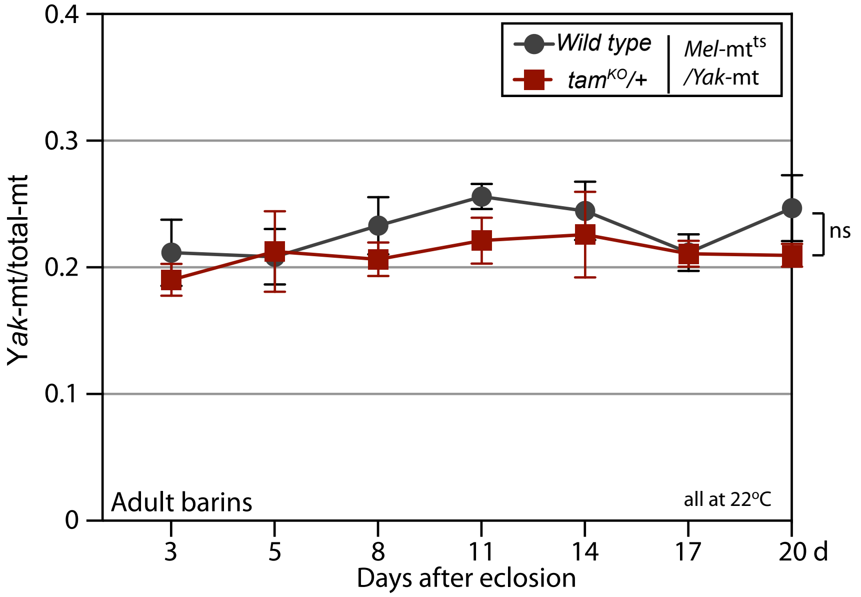

Fig. S6. Alteration in *tam* gene dose does not substantially alter heteroplasmy ratios in the absence of a disparity in gene function. A time course during adulthood shows the *Yak*-mt/total-mt ratios in brains of wild type (nuclear) heteroplasmic flies (black) and tam^{KO}/+ heteroplasmic flies (red) when the flies are raised at 22°C (a permissive temperature for $mt{:}COI^{ts}$) until the ages indicated. tam^{KO}/+ does not alter the Yak-mt/total-mt ratios. Each data point represents the mean of average of 8 biological repeats collected at each time. Error bars represent SE. No significant difference was detected in a one-way ANOVA/Tukey's multiple comparison test.

Figure S7

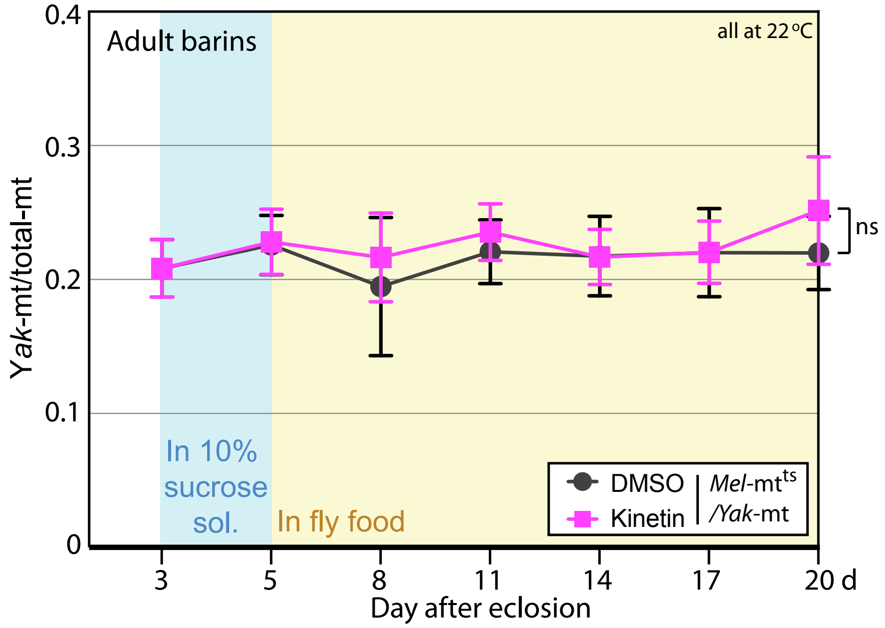

Fig. S7. Kinetin feeding does not substantially alter heteroplasmy levels in the absence of a disparity in gene function. A time course during adulthood shows the *Yak*-mt/total-mt ratios in the brains of heteroplasmic flies raised at 22°C (a permissive temperature for *mt:COI^{ts}*) that were fed (see Methods) food with solvent (DMSO) as controls (black) or food with 100 µM kinetin from day 3 (violet) until the indicated time of sampling. The kinetin feeding does not significantly modify the *Yak*-mt/total-mt ratios. Each data point represents the mean of 8 biological repeats collected at each time. Error bars represent SE. No significant difference was detected in a one-way ANOVA/Tukey's multiple comparison test.

Figure S8.

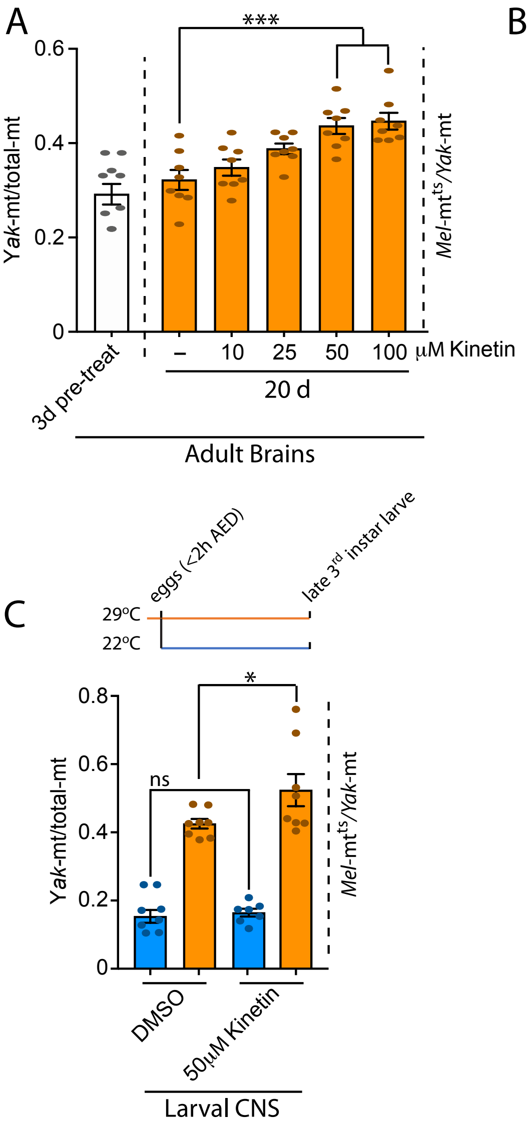

Fig. S8. Kinetin acts as a dose-dependent enhancer of mt genome quality control. (*A*) Increasing doses of kinetin fed to heteroplasmic flies from 3 days post-eclosion to 20 days improved mt genome quality. Brains were dissected from adult heteroplasmic flies (*Mel-mt^ts/Yak-mt*| *wild type*) either at day 3 (pre-treatment control) or day 20 after 17 additional days at 29°C with treatment at different concentrations of kinetin (0-100 µM, as indicated). These brains were then tested for the ratio of *Yak*-mt to total-mt. **(*B*)** Adenine suppresses the effect of the adenosine analog, kinetin. Brains dissected from adult heteroplasmic flies either at day 3 (pre-treatment control), or at day 20 after 17 additional days at 29°C with treatment with control DMSO, 100 µM kinetin, 100 µM adenine, or 100 µM kinetin+100 µM adenine. These were then were assayed for *Yak*-mt/total-mt. Adding adenine naturalized the effect of kinetin treatment on quality control of mt genomes. Tissue from 8 individuals was analyzed for each condition. **(*C*)** Kinetin action in the larvae. Heteroplasmic larvae (*Mel-mt^ts/Yak-mt*| *wild type*) were raise at either 22°C or 29°C in the presence of 50 µM kinetin or solvent control (DMSO). The ratio of *Yak*-mt/total-mt was measured in the CNS of late 3rd larvae. * = $p < 0.05$, ** = $p < 0.01$, and *** = $p < 0.001$ by one-way ANOVA/Tukey's multiple comparison test. Each data point represents the mean of average of 8 biological repeats collected at each time and treatment. Error bars represent SE

Figure S9.

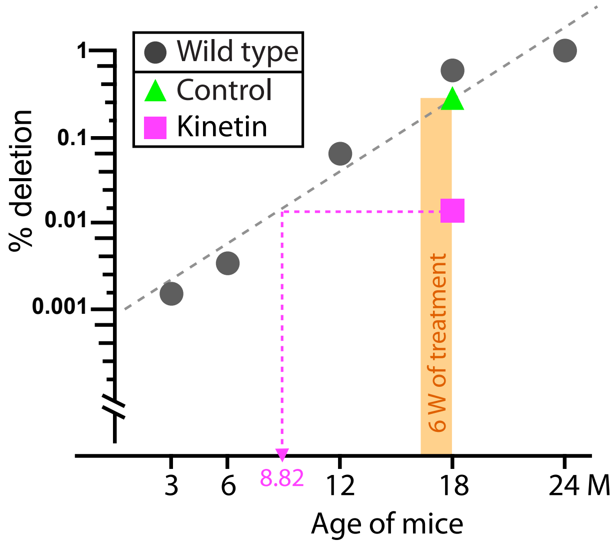

Fig. S9. Kinetin feeding reduces mutational load in C57BL6/J mice. Replotting of the data in Fig. 4*F* and 4*G* on a single graph using a logarithmic scale helps visualize the early stages of the age-associated rise in abundance of the minor-arc deletion and the effect of kinetin feeding. In control mice (grey dots), accumulation of the minor arc deletion is roughly exponential over the period of analysis (dashed grey line is a linear regression fit). Kinetin (pink ■) feeding for 6 weeks (orange bar) reduces the level of the minor-arc deletion while feeding control food does not (green Δ). Since the post-treatment level is considerably below the level expected for the onset of kinetin feeding, we suggest that kinetin selects against pre-existing mutations.
